# Prevalence of nuclear and mitochondrial DNA mutations related to adult mitochondrial disease

**DOI:** 10.1002/ana.24362

**Published:** 2015-03-28

**Authors:** Gráinne S. Gorman, Andrew M. Schaefer, Yi Ng, Nicholas Gomez, Emma L. Blakely, Charlotte L. Alston, Catherine Feeney, Rita Horvath, Patrick Yu‐Wai‐Man, Patrick F. Chinnery, Robert W. Taylor, Douglass M. Turnbull, Robert McFarland

**Affiliations:** ^1^ Wellcome Centre for Mitochondrial Research, Newcastle UniversityNewcastle upon TyneUnited Kingdom; ^2^ Institute of Neuroscience, Henry Wellcome Building for NeuroecologyNewcastle upon TyneUnited Kingdom; ^3^ Institute of Genetic Medicine, Newcastle UniversityNewcastle upon TyneUnited Kingdom

## Abstract

**Objective:**

The prevalence of mitochondrial disease has proven difficult to establish, predominantly as a result of clinical and genetic heterogeneity. The phenotypic spectrum of mitochondrial disease has expanded significantly since the original reports that associated classic clinical syndromes with mitochondrial DNA (mtDNA) rearrangements and point mutations. The revolution in genetic technologies has allowed interrogation of the nuclear genome in a manner that has dramatically improved the diagnosis of mitochondrial disorders. We comprehensively assessed the prevalence of all forms of adult mitochondrial disease to include pathogenic mutations in both nuclear and mtDNA.

**Methods:**

Adults with suspected mitochondrial disease in the North East of England were referred to a single neurology center from 1990 to 2014. For the midyear period of 2011, we evaluated the minimum prevalence of symptomatic nuclear DNA mutations and symptomatic and asymptomatic mtDNA mutations causing mitochondrial diseases.

**Results:**

The minimum prevalence rate for mtDNA mutations was 1 in 5,000 (20 per 100,000), comparable with our previously published prevalence rates. In this population, nuclear mutations were responsible for clinically overt adult mitochondrial disease in 2.9 per 100,000 adults.

**Interpretation:**

Combined, our data confirm that the total prevalence of adult mitochondrial disease, including pathogenic mutations of both the mitochondrial and nuclear genomes (≈1 in 4,300), is among the commonest adult forms of inherited neurological disorders. These figures hold important implications for the evaluation of interventions, provision of evidence‐based health policies, and planning of future services. Ann Neurol 2015 Ann Neurol 2015;77:753–759

Mitochondrial disorders are frequently multisystemic in nature and cause significant morbidity and mortality.^1^, ^2^ Unfortunately, to date, there are few effective treatments and no known cure. The overall disease burden is potentially extensive, resulting in substantial direct and indirect health care costs to the patient and society as a whole. To date, little is known of the true epidemiological burden of mitochondrial disease. Yet such information will have substantial implications for evaluation of interventions, provision of evidence‐based health policies, and planning of future services for this chronic condition.

Accurate assessment of mitochondrial disease prevalence has proven difficult in the past. Meticulous clinical and biochemical characterization of patients remains fundamental to diagnostic yield. Yet diverse and expanding clinical features, variable genotype–phenotype correlates, and the complex structure of referral pathways have all impeded attempts to gauge the true epidemiological impact of mitochondrial disease. Recent advances in diagnostic techniques in tandem with streamlining of referral pathways in the United Kingdom and deployment of extensive family tracing have improved case ascertainment and permitted, for the first time, recording of the minimum prevalence of adult mitochondrial disease, to include pathogenic mutations in both mitochondrial DNA (mtDNA) and nuclear DNA (nDNA).

## Subjects and Methods

Adult cases (>16 years old) with suspected mitochondrial disease were ascertained following referral to a single specialist mitochondrial center in the North East of England between 1990 and 2011. Referral patterns to this center are drawn mainly from tertiary neurology and neuro‐ophthalmology sources, but historical links with other specialties likely to encounter mitochondrial disease (diabetes, renal, cardiac, and audiology clinics) account for other common routes of referral. The Newcastle Mitochondrial Centre is among only 3 highly specialized, national referral centers for mitochondrial disease in the United Kingdom.

Those individuals with pathogenic mtDNA or nDNA mutations, or those with unidentified nuclear mutations, but pathological multiple mtDNA deletions evident in muscle, who were alive at the midyear period of 2011, were identified. Those with multiple mtDNA deletions in muscle were only included when there was no evidence of other muscle pathology, the mtDNA deletions had been independently verified using 2 different techniques (long‐range polymerase chain reaction [PCR], Southern blot or real‐time PCR), and the clinical and biochemical (>3% cyclooxygenase‐deficient fibers) features were consistent with a diagnosis of mitochondrial disease. Affected individuals were included if they lived within the geographical boundary of the North East of England government office region that is defined by 6 postcodes (NE, DH, DL, SR, TS, and TD) and includes County Durham, Northumberland, Newcastle upon Tyne, Sunderland, North Tyneside, Gateshead, Stockton‐on‐Tees, South Tyneside, Middlesbrough, Redcar and Cleveland, Darlington, and Hartlepool. Comprehensive pedigree analysis and family tracing was performed, identifying other clinically affected individuals and also unaffected family members at risk of disease. In contrast to our previous study,^3^ improvements in noninvasive testing^4^, ^5^ have allowed us to include elderly individuals, beyond working age, where a positive genetic diagnosis was confirmed.

### Ascertainment of Clinically Affected Adults with Disease Due to mtDNA Point Mutations, Deletions, or Duplications, or nDNA Mutations

Adopting our previous ascertainment criteria,^3^ adults exhibiting signs and symptoms consistent with a confirmed molecular genetic diagnosis of mitochondrial disease in either the individual or their pedigree were defined as clinically affected. Whenever possible, a molecular genetic diagnosis was confirmed in several tissues including blood, urine, or muscle using established quantitative techniques described in detail elsewhere.^4^, ^5^

### Ascertainment of Individuals at Risk for Development of mtDNA Disease

Comprehensive pedigree data were collected from all affected individuals, to define individuals at risk for development of mitochondrial disease. We included both adults and children, as previously reported,^3^ and again restricted our evaluation to first‐degree relatives of those patients with definite clinical disease or asymptomatic individuals known to harbor a familial pathogenic mutation of mtDNA. Molecular genetic analysis was confirmed whenever possible.

### Statistical Analysis

Population census data for the midyear period of 2011, as specified by the Office for National Statistics (ONS) of the United Kingdom,^6^ was used to derive denominators and calculate the minimum prevalence of both mtDNA and nDNA disease in adult individuals and the minimum prevalence of unaffected individuals at risk of developing mtDNA mitochondrial disease. Ninety‐five percent confidence limits were calculated for each of these groups as described elsewhere.^7^

## Results

### Prevalence of Pathogenic Mutations of Either mtDNA or nDNA

The minimum point prevalence of clinically affected adults with mitochondrial disease attributable to either the mitochondrial or nuclear genome is 12.5 in 100,000 (95% confidence interval (CI) = 11.1–14.1 × 10^−5^), whereas the prevalence of all pathogenic mutations in both nDNA and mtDNA is 23 in 100,000 (≈1 in 4,300; 95% CI = 14.6–34.5 × 10^−5^).

### Clinically Affected Individuals with mtDNA Disease

The regional adult population (>16 years old) figure for midyear 2011 was 2,134,449,^6^ representing a 2.2% increase in the prevalence data denominator over the preceding decade, since our last epidemiological evaluation of the prevalence of mtDNA disorders only. This was relative to an approximately 7% national average change in UK population size.^8^ Clinically affected adults with mtDNA mutations (n = 204) were identified, determining a minimum point prevalence of 9.6 in 100,000 (95% CI = 8.3–11.0 × 10^−5^), in this population (Table 1). Seventy‐eight adults (minimum prevalence = 3.7 × 10^−5^, 95% CI = 2.9–4.6 × 10^−5^) were affected by 1 of the 3 common Leber hereditary optic neuropathy (LHON)^9^ pathogenic mutations: m.1178G>A (n = 43, minimum prevalence = 2.0 × 10^−5^, 95% CI = 1.5–2.7 × 10^−5^), m.3460G>A (n = 29, minimum prevalence = 1.4 × 10^−5^, 95% CI = 0.9–2.0 × 10^−5^), and m.14484T>C (n = 6, minimum prevalence = 0.3 × 10^−5^, 95% CI = 0.1–0.6 × 10^−5^). The most common, pathogenic mtDNA point mutation identified was the m.3243A>G mutation (n = 74, minimum prevalence = 3.5 × 10^−5^, 95% CI = 2.7–4.4 × 10^−5^). Thirty‐one symptomatic adults harboring single large‐scale mtDNA deletions were also identified (minimum prevalence = 1.5 × 10^−5^, 95% CI = 1.0–2.1 × 10^−5^). Five individuals (minimum prevalence = 0.2 × 10^−5^, 95% CI = 0.1–0.5 × 10^−5^) were clinically affected by the m.8344A>G mutation, manifesting as myoclonic epilepsy with ragged‐red fibers (MERRF).^10^ The remaining affected adults harbored 1 of 10 other mtDNA point mutations: m.14709T>C (n = 3), m.5816A>G (n = 3), m.5650A>G (n = 2), m.8993T>C (n = 2), m.1624C>T (n = 1), m.12258G>A (n = 1), m.8993T>G (n = 1), m.12283G>A (n = 1), m.12320A>G (n = 1), and m.10010T>C (n = 1).

**Table 1:** Prevalence Estimate for mtDNA Mutations in North East England

mtDNA Mutation	Affected, No.	Prevalence in Affected Adults (95% CI)	At Risk, No.	Prevalence in Adults and Children at Risk (95% CI)
Single mtDNA deletion^a^	31	1.5 (1.0–2.1) × 10^−5^	0	0
Primary LHON mutations				
m.3460G>A, *MT‐ND1*	29	1.4 (0.9–2.0) × 10^−5^	36	1.4 (1.0–1.9) × 10^−5^
m.11778G>A, *MT‐ND4*	43	2.0 (1.5–2.7) × 10^−5^	69	2.7 (2.1–3.4) × 10^−5^
m.14484T>C, *MT‐ND6*	6	0.3 (0.1–0.6) × 10^−5^	10	0.4 (0.2–0.7) × 10^−5^
Subtotal	78	3.7 (2.9–4.6) × 10^−5^	115	4.4 (3.7–5.3) × 10^−5^
mt‐tRNA point mutations				
m.1624 C>T, *MT‐TV*	1	0.05 (0.0–0.3) × 10^−5^	0	0
m.3243A>G, *MT‐TL1*	74	3.5 (2.7–4.4) × 10^−5^	115	4.4 (3.7–5.3) × 10^−5^
m.5650A>G, *MT‐TA*	2	0.1 (0.0–0.3) × 10^−5^	3	0.1 (0.0–0.3) × 10^−5^
m.5816A>G, *MT‐TC*	3	0.1 (0.0–0.4) × 10^−5^	0	0
m.8344A>G, *MT‐TK*	5	0.2 (0.1–0.5) × 10^−5^	12	0.5 (0.2–0.8) × 10^−5^
m.10010T>C, *MT‐TG*	1	0.05 (0.0–0.3) × 10^−5^	0	0
m.12258G>A, *MT‐TS2*	1	0.05 (0.0–0.3) × 10^−5^	12	0.5 (0.2–0.8) × 10^−5^
m.12283G>A, *MT‐TL2*	1	0.05 (0.0–0.3) × 10^−5^	0	0
m.12320A>G, *MT‐TL2*	1	0.05 (0.0–0.3) × 10^−5^	4	0.2 (0.0–0.4) × 10^−5^
m.14709 T>C, *MT‐TE*	3	0.1 (0.0–0.4) × 10^−5^	19	0.7 (0.4–1.1) × 10^−5^
Subtotal	92	4.3 (3.5–5.3) × 10^−5^	165	6.3 (5.4–7.4) × 10^−5^
mt‐mRNA point mutations				
m.8993T>C, *MT‐ATP6*	2	0.1 (0.0–0.3) × 10^−5^	0	0
m.8993T>G, *MT‐ATP6*	1	0.05 (0.0–0.3) × 10^−5^	2	0.1 (0.0–0.3) × 10^−5^
Subtotal	3	0.1 (0.0–0.4) × 10^−5^	2	0.1 (0.0–0.3) × 10^−5^
Total	204	9.6 (8.3–11.0) × 10^−5^	282	10.8 (9.6–12.2) × 10^−5^

a Denotes sporadic mutations (all others are maternally inherited).

CI=confidence interval; LHON = Leber hereditary optic neuropathy; *MT‐ATP6* = mitochondrial ATP synthase F0 subunit 6; *MT‐ND1* = mitochondrial NADH dehydrogenase subunit 1; *MT‐ND4* = mitochondrial NADH dehydrogenase subunit 4; *MT‐ND6* = mitochondrial NADH dehydrogenase subunit 6; *MT‐TA* = mitochondrial tRNA alanine; *MT‐TC* = mitochondrial tRNA cysteine; *MT‐TE* = mitochondrial tRNA glutamic acid; *MT‐TG* = mitochondrial tRNA glycine; *MT‐TK* = mitochondrial tRNA lysine; *MT‐TL1* = mitochondrial tRNA leucine 1; *MT‐TL2* = mitochondrial tRNA leucine 2; *MT‐TS2* = mitochondrial tRNA serine 2; *MT‐TV* = mitochondrial tRNA valine.

### At‐Risk Individuals with mtDNA Disease

The midyear 2011 population of adults and children in North East England was 2.6 million.^6^ We identified 282 individuals at risk for the development of mtDNA disease. This equates to a minimum point prevalence of 10.7 in 100,000 (95% CI = 9.6–12.2 × 10^−5^; see Table 1).

### Clinically Affected Individuals with nDNA Disease

We identified 62 clinically affected individuals with nDNA disease, and this equates to a minimum point prevalence of 2.9 in 100,000 (95% CI = 2.2–3.7 × 10^−5^; Table 2). Recessive mutations in *SPG7* (spastic paraplegia 7) and dominant mutations in *PEO1* (progressive external ophthalmoplegia 1) were the most common pathogenic nDNA mutations identified in clinically affected individuals (*SPG7*: minimum prevalence = 0.8 × 10^−5^, 95% CI = 0.5–1.3 × 10^−5^; *PEO1*: minimum prevalence = 0.7 × 10^−5^, 95% CI = 0.4‐1.2 × 10^−5^). Mutations in *OPA1* (optic atrophy 1) caused multisystemic neuromuscular disease in 8 adults in addition to visual failure (minimum prevalence = 0.4 × 10^−5^, 95% CI = 0.2–0.7 × 10^−5^). Only autosomal recessive *POLG* (polymerase gamma) mutations were identified in the region, and these caused clinically overt disease in 6 individuals. A further 3 individuals were found to harbor a single pathogenic mutation. Dominant mutations in *RRM2B* (ribonucleotide reductase M2 B [TP53 inducible]) caused clinically overt disease in 5 adults (minimum prevalence = 0.2 × 10^−5^, 95% CI = 0.1–0.5 × 10^−5^). Only 5 clinically affected adult patients with progressive external ophthalmoplegia (PEO) and multiple mtDNA deletions in muscle remained genetically undetermined (minimum prevalence = 0.2 × 10^−5^, 95% CI = 0.1–0.5 × 10^−5^). The remaining 6 clinically affected individuals harbored mutations in the following nuclear genes: *DNM2* (dynamin 2; n = 1), *SDHA* (succinate dehydrogenase complex, subunit A; n = 2), *ETFDH* (electron‐transferring‐flavoprotein dehydrogenase; n = 1), and *TRIT1* (tRNA isopentenyltransferase 1; n = 2; see Table 2).

**Table 2:** Prevalence Estimate for Nuclear Gene Mutations in North East England

Nuclear Gene Defects	Affected, No.	Prevalence in Affected Adults (95% CI)
*SPG7*, ar	17	0.8 (0.5–1.3) × 10^−5^
*PEO1*, ad	15	0.7 (0.4–1.2) × 10^−5^
*OPA1*, ad	8	0.4 (0.2–0.7) × 10^−5^
*POLG*, ar	6	0.3 (0.1–0.6) × 10^−5^
*RRM2B*, ad	5	0.2 (0.1–0.5) × 10^−5^
*SDHA*, ad	2	0.1 (0.0–0.3) × 10^−5^
*TRIT1*, ar	2	0.1 (0.0–0.3) × 10^−5^
*DNM2*, ad	1	0.05 (0.0–0.3) × 10^−5^
*ETFDH*, ar	1	0.05 (0.0–0.3) × 10^−5^
Genetically undetermined	5	0.2 (0.1–0.5) × 10^−5^
Total	62	2.9 (2.2–3.7) × 10^−5^

ad = autosomal dominant; ar = autosomal recessive; CI = confidence interval; *DNM2* = dynamin 2; *ETFDH* = electron‐transferring‐flavoprotein dehydrogenase; *OPA1* = optic atrophy 1; *PEO1* = progressive external ophthalmoplegia 1 protein; *POLG* = polymerase gamma; *RRM2B* = ribonucleotide reductase M2 B (TP53 inducible); *SDHA* = succinate dehydrogenase complex, subunit A; *SPG7* = spastic paraplegia 7; *TRIT1* = tRNA isopentenyltransferase 1.

## Discussion

The implementation of noninvasive diagnostic methods and advances in next generation sequencing techniques continue to transform our diagnostic approach to mitochondrial disorders.^11^, ^12^ Yet despite these significant advances in our understanding of the molecular basis of mitochondrial disease, making a comprehensive diagnosis still remains a formidable task. This is in part due to the recognition of a rapidly expanding spectrum of clinical features and to the sheer diversity of genetic mutations adversely affecting mitochondrial function, emphasizing the fundamental importance of fastidious clinical and biochemical characterization of patients. Such deep phenotyping not only is important from a diagnostic perspective, but may also improve our understanding of the often complex relationship between genotype and phenotype. Understanding these mechanistic intricacies may provide the key to determining effective future treatments and guide clinical prognosis and prevention strategies for mitochondrial disease. Appropriate case ascertainment and correct disease diagnosis remain central determinants of validity in the epidemiologic evaluation of mitochondrial disease prevalence. This in turn is fundamental to assessing current and projected cost‐of‐illness needs of mitochondrial diseases for individuals and society as a whole. This is particularly relevant at present because of the development of new in vitro fertilization–based techniques^13^ that could prevent the transmission of mtDNA disease and thus reduce patient and societal disease burden.

We have endeavored to accurately document the minimum prevalence of symptomatic nDNA mutations and symptomatic and asymptomatic mtDNA mutations causing mitochondrial diseases. The inclusion of pathogenic mutations of the nDNA, as well as mutations of the mtDNA, represents a significant evolution of our understanding of the social impact of these heterogeneous disorders. As a whole, mitochondrial disorders are among the commonest inherited neurological disorders. As individual entities, however, each genotype or phenotype represents just one facet of extremely rare disorders that pose difficult challenges in recognition, diagnosis, and ultimately treatment.

The minimum point prevalence figure for all clinical disease due to mtDNA mutations in this study is comparable with our previously published point prevalence of ≈1 in 5,000^3^; reflecting the stability of mtDNA disease prevalence both geographically and over time. This also reflects the relative stability of the population of the North East, where ONS census data^8^ showed only a 2.2% increase in the regional population numbers compared to a 7% increase in national figures in the intervening decade. The m.3243A>G mutation remains the most prevalent pathogenic mtDNA point mutation, being found in 25 pedigrees with a point prevalence of 7.8/100,000, comparable to that of in‐region figures for Mendelian‐inherited facioscapulohumeral muscular dystrophy.^14^ The prevalence of LHON remains stable; this is the most prevalent mtDNA disorder overall (point prevalence for affected LHON mutations of 3.65 per 100,000; carrier frequency of the 3 common LHON mutations of 4.42 per 100,000, with familial clustering evident, m.11778G>A: 12 pedigrees, m.3460G>A: 5 pedigrees, m.14484T>C: single pedigree; see Table 1). MERRF related to the m.8344A>G mutation remains a rare form of mitochondrial disease (0.7/100,000) in this region, similar in frequency to the m.14709T>C^15^, ^16^ mutation (0.8/100,000). Thirty‐one affected individuals with single large‐scale deletions of mtDNA were identified. All were considered to be predominantly sporadic events with no at‐risk individuals identified. However, we do acknowledge that maternal transmission may occur,^17^ and the advent of a prediction tool,^18^ providing guidance to the patient and clinician about prognosis and risk of transmission, may alter this approach in the future. We identified 2 other pathogenic mutations, in at‐risk individuals. These included 2 mtDNA point mutations, m.13094T>C and m.4300A>G, in whom the index cases were deceased at the time of analysis but other at‐risk family members were known to live in region. These data exemplify the importance of fastidious family tracing and continued clinical surveillance.

Mutations in nDNA genes affect function of the mitochondrial respiratory chain in a variety of ways, including: synthesis, assembly, or chaperoning of individual components of the mitochondrial respiratory chain; synthesis, maintenance, repair, and translation of mitochondrial DNA; and the processes of mitochondrial fission and fusion, which maintain mitochondrial networks. Nuclear mutations account for overt disease in 2.9 per 100,000 of the adult population in the North East of England, approximately one‐third of the prevalence for mtDNA mutations. To date, 14 nuclear encoded genes, *TYMP* (thymidine phosphorylase), *SLC25A4* (solute carrier family 25 [mitochondrial carrier; adenine nucleotide translocator], member 4), *POLG*, *PEO1*, *OPA1*, *POLG2*, *RRM2B*, *TK2* (thymidine kinase 2), *DGUOK* (deoxyguanosine kinase), *MPV17* (mitochondrial inner membrane protein), *MGME1* (mitochondrial genome maintenance exonuclease), *DNA2* (DNA replication helicase/nuclease 2), *SPG7*, and *AFG3L2* (AFG3‐like AAA ATPase 2),^19^, ^20^ have been reported to be associated with ophthalmoparesis and multiple mtDNA deletions in muscle in adult onset PEO patients. Notably, mutations in *SPG7* (≈4/100,000) and *PEO1* (≈3/100,000), followed by *OPA1* (≈1/100,000) and *RRM2B* (≈0.9/100,000) have emerged as major causes of adult mitochondrial disease. Recessive mutations in *POLG* (0.6/100,000) are also emerging as an important cause of mitochondrial disease in the North East of England. Delineation of an at‐risk group of individuals harboring single pathogenic mutations in *POLG* is important, even in nonconsanguineous families. Given the relatively common prevalence of pathogenic *POLG* mutation heterozygosity in European populations and the potential severity of the phenotype due to recessive mutations, the provision of genetic counseling to individuals and their partners should be made, even where the risks to future offspring are small. Less than 10% of adult patients with PEO and muscle restricted mtDNA deletions remain genetically undetermined, reflecting recent advances in diagnostic yield from implementation of next generation sequencing and deep clinical phenotyping of all patients attending our center.

Inherent difficulties exist in the calculation of those at risk of nDNA‐related mitochondrial disease because of the heterogeneous nature of inheritance patterns of nDNA mutations, variability in disease penetrance and clinical expression, and with the recent advent of next generation sequencing, a recognition of the rapidly expanding number of nDNA mutations causing mitochondrial disease. Accepting such limitations, and implementing the same criteria to determine individuals at risk of mtDNA‐related mitochondrial disease, we would conservatively estimate that a minimum of 5.9 in 100,000 (154 individuals; 95% CI = 5.0–6.9 × 10^−5^) are potentially at risk of nDNA‐related mitochondrial disease (Supplementary Table).

Comparison with point prevalence figures for mitochondrial disorders in other regions remains difficult. These current results for mtDNA mutations are similar to that reported previously in adult patients with mitochondrial disease in the North East of England a decade earlier and as such act as comparative data.^3^ Although the frequency of mtDNA mutations remain stable in our patient cohort, the point prevalence for the m.3243A>G mutation remains significantly lower than the frequency reported by Majamaa et al.^21^ The reasons for this remain unclear. However, given that the point prevalence figures remain stable in the intervening decade in this English cohort, the genetic background and population structure may play a more significant role than study design in creating variability in prevalence rates than originally purported.^3^ In addition, the impact of familial clustering on prevalence frequency is more marked in the rare forms of both mtDNA and nDNA and may serve as a confounding factor of the estimated minimum prevalence rates of these extremely rare disorders. The almost homogenous white English ethnic profile and lower rates of parental consanguinity in the North East of England compared with the ethnically diverse conurbations of North, Central, and East Midlands and South East England will also influence the prevalence rates we have observed.^22^ This is particularly pertinent to pediatric mitochondrial disease, where recessive mutations in nDNA are more often the genetic etiology.

It is clear from this detailed population study of adult patients with mitochondrial disease that physicians recognize the significant risk of disease posed to individuals who have family members diagnosed with transmissible forms of mitochondrial disease. Moreover, we would suggest that family history alone serves as a surrogate marker for the development of mtDNA‐related mitochondrial disease.

### Conclusion

This rigorous cross‐sectional study benefiting from improved case ascertainment and diagnosis over the past decade indicates that adult mitochondrial diseases are among the most prevalent groups of inherited neurological disorders, affecting up to 1 in 4,300 adults in the United Kingdom. Although we have documented a relatively stable prevalence rate for mtDNA mutations over time, it seems likely that an improved understanding of nuclear phenotypes and advances in next generation sequencing technologies including whole exome and whole genome sequencing are likely to lead to an increased recognition of mitochondrial disease attributable to mutations of the nuclear genome. However, given that <10% of nuclear phenotypes in this cohort remain genetically undetermined, this figure is unlikely to change significantly, unless the perceived phenotypic spectrum is also expanded. Combined, these findings will facilitate more robust assessment of the level of services and financial investment required to provide early detection, prevention, and treatment of mitochondrial diseases.

## Authorship

G.S.G. and A.M.S. are joint first authors.

## Potential Conflicts of Interest

Nothing to report.

## Supporting information

Supporting Information Table 1.
