# Supplementary material for: Prevalence of nuclear and mitochondrial DNA mutations related to adult mitochondrial disease
Source: Ann Neurol. 2015 Mar 28;77(5):753–9. doi: 10.1002/ana.24362 (PMC4737121; doi:10.1002/ana.24362)
Supplement: Supplementary file 1 — Supporting Information Table 1. [file ANA-77-753-s001.docx]

| **Nuclear Genes with Mutations** | **At risk**  **(n=)** | **Prevalence in Adults and Children ‘at risk’ (95% CI)** |
| --- | --- | --- |
| *PEO1* | 59 | 2.3 (1.7-2.9) x 10^-5^ |
| *POLG (AR)* | 8 | 0.3 (0.1-0.6) x 10^-5^ |
| *RRM2B* | 17 | 0.7 (0.4-1.0) x 10^-5^ |
| *OPA1* | 18 | 0.7 (0.4-1.1) x 10^-5^ |
| *SDH* | 5 | 0.2 (0.1-0.4) x 10^-5^ |
| *SPG7* | 33 | 1.3 (0.9-1.8) x 10^-5^ |
| *DNM2* | 4 | 0.2 (0.0-0.4) x 10^-5^ |
| *ETFDH* | 1 | 0.05 (0.0-0.2) x 10^-5^ |
| *TRIT1* | 1 | 0.05 (0.0-0.2) x 10^-5^ |
| Unknown | 8 | 0.3 (0.1-0.6) x 10^-5^ |
| **TOTAL** | **154** | **5.9 (5.0-6.9)** x 10^-5^ |

**Supplemental table 1 Prevalence estimate for ‘at risk’ individuals with nuclear gene** **mutations in North East England**

CI= confidence interval; AD= autosomal dominant; AR= autosomal recessive; n=number; *PEO1= Progressive External Ophthalmoplegia 1 Protein; POLG = polymerase gamma; RRM2B= Ribonucleotide Reductase M2 B (TP53 Inducible); OPA1= Optic Atrophy 1; SPG7= Spastic Paraplegia 7; SDHA= succinate dehydrogenase complex, subunit A; DNM2= dynamin 2; ETFDH****=*** *Electron-Transferring-Flavoprotein Dehydrogenase; TRIT1= tRNA isopentenyltransferase 1*
